# microRNA regulatory circuits in a mouse model of inherited retinal degeneration

**DOI:** 10.1038/srep31431

**Published:** 2016-08-16

**Authors:** Arpad Palfi, Karsten Hokamp, Stefanie M. Hauck, Sebastian Vencken, Sophia Millington-Ward, Naomi Chadderton, Mathew Carrigan, Elod Kortvely, Catherine M. Greene, Paul F. Kenna, G. Jane Farrar

**Affiliations:** 1 Smurfit Institute of Genetics, Trinity College Dublin, Dublin 2, Ireland; 2 Research Unit Protein Science, Helmholtz Zentrum Munchen - German Research Center for Environmental Health (GmbH), Neuherberg, Germany; 3 Respiratory Research Division, Dept. Medicine, Royal College of Surgeons in Ireland, Education and Research Centre, Beaumont Hospital, Dublin 9, Ireland; 4 Institute of Ophthalmic Research, Center for Ophthalmology, University of Tubingen, Tubingen, Germany

## Abstract

miRNA dysregulation is a hallmark of many neurodegenerative disorders, including those involving the retina. Up-regulation of miR-1/133 and miR-142, and down-regulation of miR-183/96/182 has been described in the RHO-P347S mouse retina, a model for a common form of inherited blindness. High-throughput LC-MS/MS was employed to analyse the protein expression of predicted targets for these miRNAs in RHO-P347S mouse retinas; 133 potential target genes were identified. Pathway over-representation analysis suggests G-protein signaling/visual transduction, and synaptic transmission for miR-1, and transmembrane transport, cell-adhesion, signal transduction and apoptosis for miR-183/96/182 as regulated functions in retina. Validation of miRNA-target mRNA interactions for miR-1, miR-96/182 and miR-96 targeting Ctbp2, Rac1 and Slc6a9, respectively, was demonstrated *in vitro*. *In vivo* interaction of miR-183/96/182 and Rac1 mRNA in retina was confirmed using miR-CATCH. Additional miRNAs (including miR-103-3p, miR-9-5p) were both predicted to target Rac1 mRNA and enriched by Rac1-miR-CATCH. Other Rac1-miR-CATCH-enriched miRNAs (including miR-125a/b-5p, miR-378a-3p) were not predicted to target Rac1. Furthermore, levels of ~25% of the retinal Rac1 interactors were determined by LC-MS/MS; expression of Rap1gds1 and Cav1 was elevated. Our data suggest significant utilisation of miRNA-based regulation in retina. Possibly more than 30 miRNAs interact with Rac1 in retina, targeting both UTRs and coding regions.

MicroRNAs (miRNAs) are small (~22-nt in mature form) non-coding RNAs involved in post-transcriptional regulation of gene expression in eukaryotes and viruses^1^. In animals, regulation via miRNAs plays an essential role in control of gene expression. Approximately 1000 miRNAs have been identified in humans^2^. It is estimated that more than 60% of protein coding genes in humans are regulated by miRNAs^3^. *In silico* predictions reveal that each miRNA can in principle target many (hundreds) mRNAs, while individual mRNAs can also be targeted by many miRNAs^3^,^4^. miRNAs are involved in many physiological and pathophysiological processes including development, apoptosis and cancer^1^,^5^,^6^. Some 250 miRNAs are expressed in the retina where they are components of a complex gene regulatory network^7^,^8^,^9^,^10^. Key processes regulated by miRNAs in retina include adaptation to differing light intensities, rapid turnover of the phototransduction cascade and maintenance of cellular homeostasis under high activity^8^,^9^. Furthermore, miRNAs are implicated in control of cell differentiation, maturation and survival in retina^9^,^11^.

Similar to many other human conditions^6^, retinal disease involves miRNA dysregulation. Indeed our prior work highlighted a common miRNA signature^12^ in four mouse models of retinitis pigmentosa (RP), including down-regulation of miR-183, miR-96 and miR-182, and up-regulation of miR-1, miR-133, and miR-142. A different miRNA profile was observed in a canine model of X-linked progressive retinal atrophy; highly up-regulated miRNAs included miR-146a, miR-19a, miR-21 and miR-101^13^. Other retinal disorders, such as glaucoma^14^ are also characterized by miRNA dysregulation.

The genes encoding miR-183/96/182 are clustered within 4 kb on mouse chr6qA3. This cluster is referred to as ‘sensory organ-specific’, is highly expressed in retina and regulated by light^8^,^15^. The functional significance of the miR-183/96/182 cluster in retina has been explored. Inactivation of the cluster led to photoreceptor synaptic defects, electroretinography (ERG) abnormalities and progressive retinal degeneration (RD) in mice^16^. Depletion of miRNAs from cones resulted in loss of cone outer segments but was reversed by re-expression of just two miRNAs; miR-183 and miR-182^17^. While the role of miR-1, miR-133, and miR-142 in retina and RD is ill defined, previous work in RP mouse models^12^ and a Müller cell ablation/RD model^10^ found significant up-regulation of these miRNAs.

There is clear evidence of a link between retinal dysfunction (disease or experimentally induced) and altered miRNA expression. Far less is known about the target genes, which are post-transcriptionally regulated by these miRNAs in retina and indeed elsewhere. As mentioned above, prior work highlighted miR-1, miR-133, miR-142 and miR-183/96/182 dysregulation in RP models^12^ including the RHO-347+/−Rho+/−(R347) mouse^18^,^19^. In the current study, the R347 mouse^18^,^19^ is further explored to probe the miRNA regulatory pathways in RP. The strategy adopted involved analysing the retinal proteome in R347 versus 129 wild type (wt) retinas via high-throughput liquid chromatography-mass spectrometry (LC-MS/MS) and predicting candidate miRNA-target mRNA pairs by matching proteins with significantly (and inversely) altered expression to *in silico* computed targets for the above six miRNAs. An interesting validated target, i.e. Rac1, previously implicated in retinal degeneration^20^,^21^,^22^, was further probed utilizing miRNA capture affinity technology (miR-CATCH) and analysis of the retinal Rac1 interactome.

## Results

### *In Silico* Target Selection

Altered expression of miR-1, miR-133, miR-142 and miR-183/96/182 in the R347 mouse model has been observed^12^. Notably, the genotype of the R347 mouse used in this study, i.e. RHO-347+/−Rho+/− (one mutant and one wt rhodopsin allele) reflects a typical genotype of a patient with autosomal dominant RP. Our miRNA target prediction pipeline used three miRNA target site prediction alogrithms (Table 1) and we filtered for targets predicted by at least two of these prediction methods (Table 1). Additionally, we accepted only those miR targets sites where the prediction tools predicted the given site at the same location (Overlapping predicted target sites in Table 1). We identified 5301 candidate targets for the above miRNAs (Table 1); 3721 unique genes, as some genes were targeted by multiple miRNAs (Table 1). To estimate the number of predicted targets, which were expressed in retina, a wt mouse retinal transcriptome library (Supplementary Table) was collated^11^,^23^. Expression values ranged between 0 and 23554 FPKM (fragments per kb of transcript per million reads) and RPKM (reads per kb of transcript per million reads). 14335 (of 22788) genes had an expression value ≥0.5 FPKM/RPKM in at least one dataset and were arbitrarily deemed as being expressed in retina; however, only 12758 of these coded for proteins (BioMart, Ensembl version 79, http://www.biomart.org). The intersection between the retinal transcriptome and predicted miRNA targets was 4718 genes (3262 unique genes; Table 1).

### Proteome Analysis of R347 versus wt Retinas

**Figure 1:**
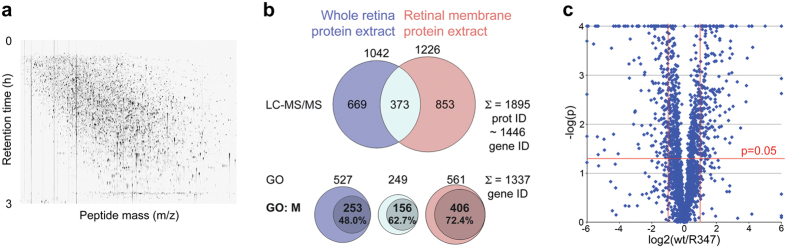
LC-MS/MS retinal proteome analysis in R347 versus wt mice. Whole retina protein (n = 4) and retinal membrane protein (n = 4) extracts were prepared from one month-old R347 and wt mice. LC-MS/MS analysis was performed on an Ultimate3000 nano HPLC system coupled to a LTQ OrbitrapXL mass spectrometer. (**a**) Representative LC-MS/MS profile detected in the retinal samples. (**b**) Distribution of the 1895 identified protein IDs, which were mapped to 1446 gene IDs. 1337 gene IDs were present in the GO database (GO); GO: M refers to entries with membrane in their GO search terms. (**c**) Volcano plot representation of the identified protein IDs in R347 versus wt retinas. X-axis indicates difference in expression level on a log2 scale, whereas the y-axis represents corresponding p-values (Student’s t-Test) on a negative log scale. Red lines indicate 0.5-fold and 2-fold differences in protein expression and significance level of p = 0.05, respectively. Scaling was limited to -6 and 6 on the horizontal axis and 4 on the vertical axis. Entries outside the limiting values were set to the corresponding limiting values.

Label-free LC-MS/MS was employed to quantify protein levels in one-month old R347 and wt mouse retinas (Fig. 1a). 1042 and 1226 protein IDs were identified from whole retina (n = 4) and retinal membrane extracts (n = 4), respectively (Fig. 1b; raw data is given in Supplementary Table S2). The combined number of identified unique protein IDs was 1895; these were mapped to 1446 MGI gene IDs using BioMart (Ensembl version 79, http://www.biomart.org); 1237 (~86%) of these genes were mapped to the retinal transcriptome library (Supplementary Table S1)^11^,^23^. Therefore it is estimated that the study enabled quantitative analysis of ~9.7% (1237/12758) of the retinal protein coding transcriptome at the protein level. Marginally greater coverage was observed in the Gene Ontology (GO) database with 1337 of the 1446 gene IDs being present in the GO database (http://geneontology.org); 72.4% of proteins from retinal membrane protein extracts had ‘membrane’ among their GO terms (Fig. 1b). Expression of 811 protein IDs was significantly different (p < 0.05) between R347 and wt samples (Fig. 1c; raw data is given in Supplementary Table S2); further analysis was undertaken as detailed below.

**Figure 2:**
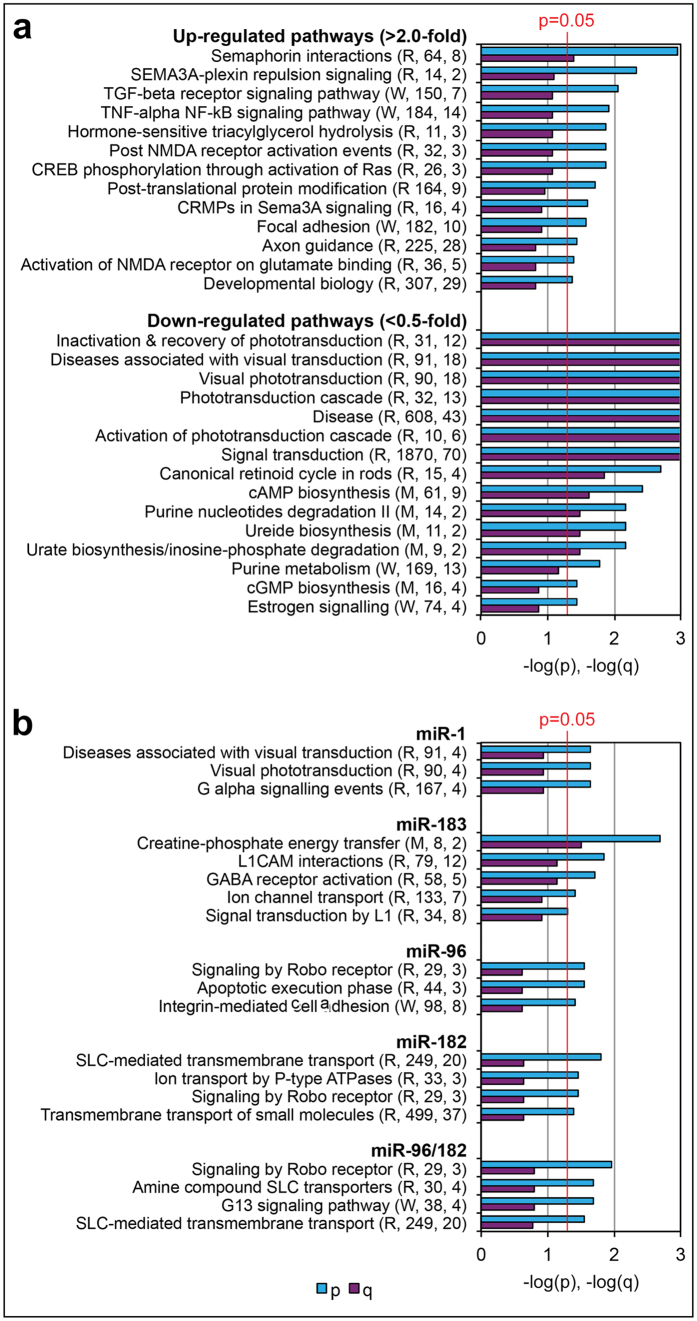
Pathway over-representation analysis in R347 versus wt retinas. (**a**) Pathways with altered protein expression in R347 versus wt retinas. Up-regulated and down-regulated protein expression levels were defined as >2.0-fold (p < 0.05) and < 0.5-fold (p < 0.05) change in expression between R347 versus wt mouse retinas, respectively. (**b**) Putative miR-1- and miR-183/96/182-regulated pathways in retina. The pathway over-representation tool in ConsensusPathDB^24^ was employed to analyse 1895 LC-MS/MS-identified retinal proteins (**a**) and 133 potential miRNA targets (**b**). The minimal overlap with the input list and the p value cut off were set to 2 and 0.05, respectively. p and q values are given for identified pathways in bar charts (scaling was limited to 3; values higher than 3 were set to 3); a red line indicates the significance level of p = 0.05. Corresponding database sources (R: Reactome; W: Wikipathways; M: MouseCyc), and absolute and effective set sizes are given in brackets with each pathway.

Pathway over-representation analysis^24^ was performed on the 1895 identified retinal proteins. First, proteins with increased (>2.0-fold) or decreased (<0.5-fold) expression between R347 and wt samples were identified (p < 0.05). Then the two groups of proteins were probed against the complete list of identified proteins; 13 (37 proteins) and 15 (44 proteins) enriched pathway-based sets were identified (Fig. 2a). Pathways with up-regulated proteins included semaphorin, TGF-beta- and TNF-alpha/NF-kB signaling, NMDA receptor-/postsynaptic activation and axon guidance, amongst others (Fig. 2a). Most but not all pathways displaying down-regulated proteins were involved in visual transduction (Fig. 2a).

### miRNA Targets

Data from *in silico* miRNA target predictions (5301 target genes, Table 1) and retinal proteome analysis (1895 proteins/1446 genes, Fig. 1) were combined. The intersection between datasets was 538 genes (372 unique genes). Of the 5301 predicted miRNA targets, 4718 were believed to be expressed in retina (Table 1), i.e. 11.4% (538/4718) of the predicted retinal target genes were analysed at the protein level. Of the 538 (372 unique) genes from the intersecting list, 248 (169 unique) genes exhibited altered expression (p < 0.05) in R347 versus wt retinas. As miRNAs suppress gene expression, a key factor in identifying potential miRNA-target mRNA pairs was an inverse relationship between levels of miRNAs and their targets. Proteins for 133 (102 unique) target genes met this criterion (p < 0.05) in either whole retina and/or retinal membrane protein samples (Supplementary Table S3). Specifically, 23, 10, 6, 18, 35 potential target genes were identified for miR-1, miR-133, miR-142, miR-183, miR-96 and miR-182, respectively (Supplementary Table S3). Considerable overlap among miR-183, miR-96 and miR-182 target genes was observed; e.g. 21 targets were potentially co-targeted by miR-96 and miR-182 (Supplementary Table S3). Pathway over-representation analysis^24^ of miRNA-specific target gene lists identified a number of enriched pathway-based sets for miR-1 and miR-183/96/182 (Fig. 2b).

**Figure 3:**
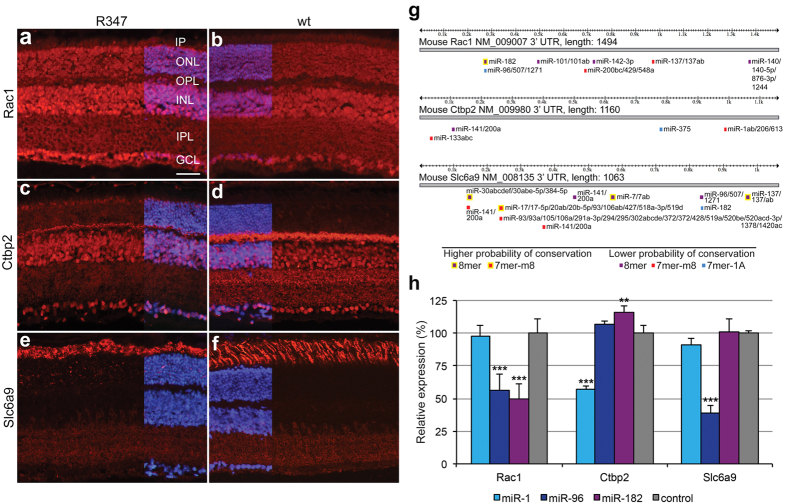
Retinal protein expression and 3′UTR assay of Rac1, Ctbp2 and Slc6a9. Cy3-labeled immunohistochemistry was performed for Rac1 (**a**,**b**), Ctbp2 (**c**,**d**) and Slc6a9 (**e**,**f**) in retinal cryosections (12 μm) of one month-old R347 and wt mice (n = 3). DAPI was used for nuclear counterstaining; DAPI signals were overlaid only on part of each image to enable better visualization of the Cy3 label. Scale bar represents 25 μm (panel (**a**)). GCL: ganglion cell layer, INL: inner nuclear layer, IPL: inner plexiform layer, ONL: outer nuclear layer, OPL: outer plexiform layer, PS: photoreceptor segment layer. (**g**) Broadly conserved miRNA target sites in the Rac1, Ctbp2 and Slc6a9 3′UTRs identified by TargetScan^4^. (**h**) Mouse Rac1, Ctbp2 and Slc6a9 3′UTR sequences in dual firefly luciferase/Renilla luciferase expression vectors and synthetic pre-miRNAs for mmu-miR-1a-3p (miR-1), mmu-miR-96-5p (miR-96), mmu-miR-182-5p (miR-182) and negative control were co-transfected into Hela cells (n = 5). 24 h post-transfection, luciferase activity of the cells was evaluated using a Dual-glow luciferase assay system. Luciferase expression levels in cells co-transfected with the negative control pre-miRNA were taken as 100% **p < 0.01, ***p < 0.001.

A subset of potential targets was selected for further analysis. Retinal expression of Rac1, Ctbp2 and Slc6a9 (Fig. 3a–f), as well as, Api5, Arcn1, Cav1, Dgke, Flot2, Igf1r, Negr1 and Tagln3 (Supplementary Figure S1), was analysed using immunohistochemistry in R347 and wt mice. Given that the R347 phenotype involves photoreceptors, the objective was to select targets with expression in this cell type. Some targets were not localized to photoreceptors; e.g. Cav1 was expressed largely in glial and vascular cells, Igf1r in immune cells and Dgke in the inner retina (Supplementary Figure S1). Three target proteins were selected for further exploration; Rac1, Ctbp2 and Slc6a9.

Immunohistochemistry revealed that Rac1, in line with prior observations^25^, was expressed in all retinal layers and had similar pattern of expression in R347 and wt retinas (Fig. 3a,b). Of the six miRNAs modulated in R347 retinas^12^ the Rac1 3′UTR contains a predicted combined target site for miR-96/182 (Fig. 3g) and a site for miR-142 (Fig. 3g). miR-96 and miR-182 levels were ~50% lower (Table 2)^12^, while Rac1 protein levels were markedly higher (~3.4-fold, LC-MS/MS) in whole retina samples in R347 versus wt mice (Table 2), suggesting a potential miR-96/miR-182-Rac1 mRNA regulatory axis. In contrast, levels of Rac1 protein in retinal membrane protein extracts were reduced by ~40% (Table 2), perhaps due to regulation via miR-142. As the increase in Rac1 protein level in whole retina samples was more significant, we opted to further analyse the miR-96/miR-182-Rac1 mRNA regulatory axis.

Ctbp2 was expressed in all retinal layers; in photoreceptors, Ctbp2 was largely confined to synapses in the outer plexiform layer (Fig. 3c,d). There was an apparent decrease in Ctbp2 in the plexiform layer in R347 versus wt retina (Fig. 3c,d). Of the six miRNAs of interest, the Ctbp2 3′UTR contains predicted target sites for miR-1 and miR-133 (Fig. 3g), the levels of which were significantly increased in R347 versus wt retinas (Table 2)^12^. Ctbp2 protein in R347 versus wt retinas was decreased by ~50% (Table 2, LC-MS/MS) suggesting that miR-1 and miR-133 may target Ctbp2; the potential miR-1-Ctbp2 mRNA interaction was further tested in the study.

Slc6a9 was highly expressed in photoreceptor outer segments, while lower expression was determined in the inner retina (Fig. 3e,f). Lectin PNA labeling co-localised with Slc6a9, indicating that Slc6a9 is expressed exclusively in cones (Supplementary Figure S2). A similar pattern of expression of Slc6a9 was observed in R347 and wt retinas; though the photoreceptor outer segments were shorter and less organized in R347 retinas (Fig. 3e,f). Of the six miRNAs of interest, the Slc6a9 3′UTR contains a predicted combined target site for miR-96/182 (Fig. 3g); the level of these miRNAs was decreased in R347 versus wt retinas (Table 2)^12^. As the protein level of Slc6a9 was increased by ~70% in R347 versus wt retinas (Table 2), the data predicted that miR-96 and miR-182 may target Slc6a9.

To evaluate if predicted miRNA-mRNA target site interactions were functional, 3′UTR assays for Rac1, Ctbp2 and Slc6a9 were performed (n = 5, Fig. 3h). Pre-miRNAs reduced luciferase expression from 3′UTR constructs with corresponding predicted target sites by ~50–60% (Fig. 3h), except for the miR-182-Slc6a9 3′UTR combination, which showed no suppression (Fig. 3h). In contrast, pre-miRNAs had no effect on luciferase expression from 3′UTR constructs, which did not contain corresponding predicted target sites (Fig. 3h).

### *In vivo* miRNA-Rac1 mRNA Interactions

**Figure 4:**
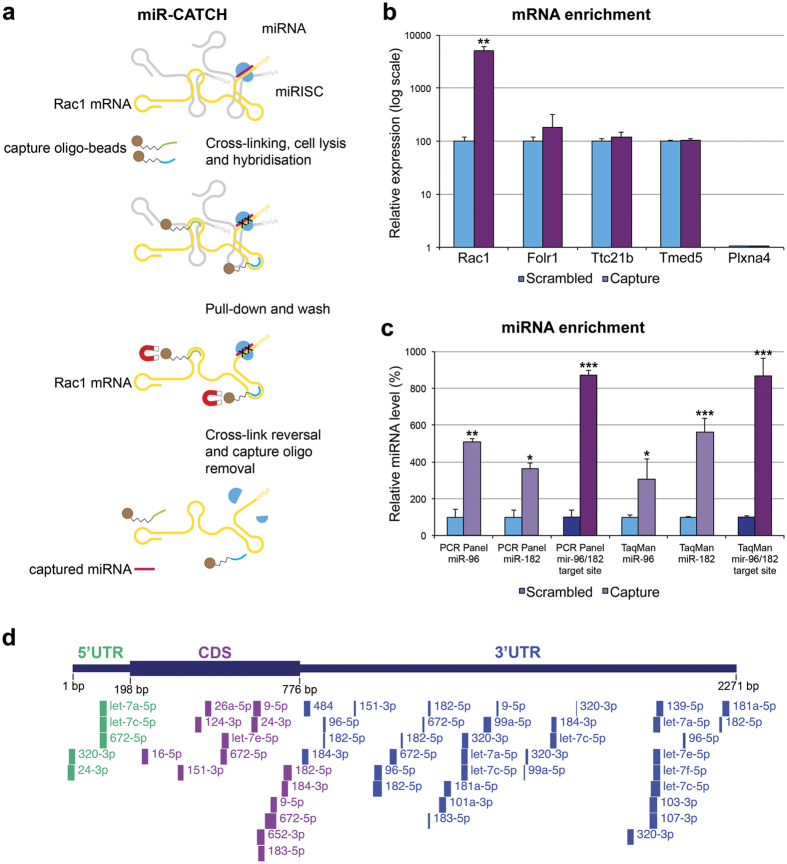
*In vivo* Rac1-miR-CATCH. (**a**) Schematic representation of miR-CATCH^24^. First, active mRNA:miRISC complexes are cross-linked using formaldehyde fixation. Cells are lysed and capture oligonucleotide probes (complexed with metal beads) are hybridized to target mRNAs of interest. Next, captured mRNAs with bound miRISC complexes are pulled down using magnetic separation. Unbound mRNAs are washed away resulting in enrichment of target mRNA:miRISCs complexes. Finally, cross-links are reversed and capture oligonucleotides removed enabling evaluation of target mRNA and the captured targeting miRNAs. (**b**) Rac1-miR-CATCH was performed using C9 plus C10 capture oligonucleotides (Capture; n = 3) or scrambled control oligonucleotide (Scrambled; n = 3). Total RNA was purified from the samples and Rac1, Ttc21b, Folr1, Tmed5 and Plxna4 mRNAs were quantified by RT-qPCR (n = 3); note that the y-axis is in log scale. (**c**) Expression of miR-96 and miR-182 was analysed using Exiqon rodent miRNA PCR panel (PCR Panel; n = 2) and Applied Biosystem TaqMan microRNA Assays (TaqMan; n = 3). As miR-96 and miR-182 target the same site (TargetScan)^4^, the combined miR-96 and miR-182 levels are also given (miR-96/182 target site). *p < 0.05, **p < 0.01, ***p < 0.001. (**d**) miRNA targeting of Rac1 mRNA was analysed via combination of *in vivo* Rac1-miR-CATCH miRNA enrichment (Exiqon’s rodent miRNA PCR panel) and *in silico* predictions for miRNAs targeting the Rac1 3′UTR (miRSystem)^29^ and the Rac1 cDNA (RNA22)^30^. Position of target sites corresponding to miRNAs, which were both enriched and *in silico* predicted to target Rac1 are given. The blocks represent the seed/target regions reported by the different prediction tools used and range in size from 6 bp to 30 bp. CDS: coding sequence.

Based on the potential involvement of Rac1 signaling in RD 20,21,22,26, the miR-96/182-Rac1 regulatory system was investigated further. Rac1 mRNA levels were determined using RT-qPCR in R347 and wt retinas and were found to be similar; 96.0 ± 2.8% and 100.0 ± 1.1%, respectively. The functional interaction between miR-96/182 and Rac1 mRNA was evaluated using *in vivo* miR-CATCH (Fig. 4a)^27^. The highest ranked off-targets for capture probes (C9 and C10) were identified using Nucleotide Blast^28^. Top rated off-targets (Ttc21b, Folr1, DXBay18, Gm14685, Cmya5, Cdh17 for C9 and Tmed5, Plxna4, Cyp2d34, Swi5, H2-Q7, H2-Q10 for C10) were analysed for miRNA target sites using TargetScan^4^ but none of these mRNAs contained predicted target sites for miR-96/182.

*In vivo* Rac1-miR-CATCH (Fig. 4a) was undertaken on pooled retinal samples from seven wt mice per group (n = 3). A ~50-fold enrichment (Fig. 4b) of Rac1 mRNA in miR-CATCH capture compared to scrambled control samples was achieved. Ttc21b and Folr1 (highest scored off-targets for C9), and Tmed5 and Plxna4 (highest scored off-targets for C10) were quantified by RT-qPCR; none of these mRNAs were enriched significantly (Fig. 4b). miRNA levels in miR-CATCH samples were analysed using the rodent miRNA PCR panel (n = 2;. Fig. 4c, raw data is given in Supplementary Table S4) and TaqMan microRNA assays (n = 3; Fig. 4c). The level of both miR-96 and miR-182 increased significantly (~3-5-fold) in Rac1-capture versus scrambled control samples using both detection techniques (Fig. 4c). As miR-96 and miR-182 target the same conserved target site (TargetScan)^4^ at the Rac1 3′UTR (Fig. 3g), the corresponding miRNA enrichment values were summed to reflect combined usage of the miR-96/182 target site, which was ~8.7-fold with both detection methods (Fig. 4c). In order to identify additional miRNAs targeting Rac1, *in silico* target site predictions for Rac1 3′UTR using miRSystem^29^ and for Rac1 cDNA (BC003828.1) using RNA22 analysis^30^ were combined with Rac1-miR-CATCH miRNA enrichment data. Apart from the conserved miR-96/182 site, two additional miR-96, four additional miR-182 (combined and unique) and two miR-183 sites were identified for the miR-183/96/182 cluster (Fig. 4d and Table 3a). In addition, a number of candidate miRNAs, which were both predicted to target Rac1 and were enriched in Rac1-miR-CATCH were found, e.g. miR-103-3p, let-7a/c/e/f-5p, miR-320-3p, miR-9-5p, miR-26a-5p, miR-151-3p (Fig. 4d and Table 3a). Notably, *in silico* prediction tools had not predicted a number of miRNAs to target Rac1 (e.g. miR-125a/b-5p, miR-378a-3p and miR-204-5p), yet they were enriched in Rac1-miR-CATCH capture versus control samples (Table 3b).

### Retinal Rac1 Interactome

**Figure 5:**
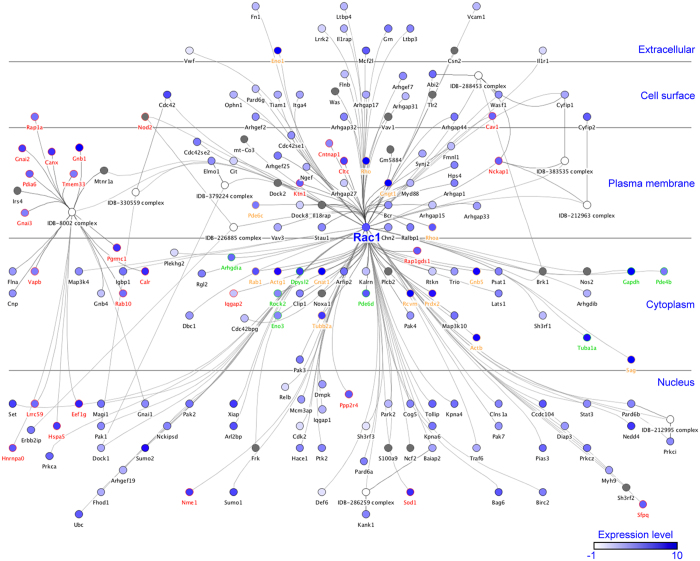
Retina Rac1 interactome. The mouse Rac1 interactome was generated in InnateDB^31^. Relative retinal expression levels determined in previous studies^11^,^23^ (Supplementary Table S1) were added in log2 of FPKM/RPKM and are depicted as fill-in shades of blue. 0.5 FPKM/RPKM was arbitrarily selected as cut-off point for positive expression (−1 on the log2 scale); symbols for proteins with less than 0.5 FPKM/RPKM in retina are depicted with grey fill-in shade. Proteins detected by LC-MS/MS in our study are highlighted in red. Mouse orthologs of Rac1 interactors previously identified in photoreceptor outer segments^33^ were added to the interactome and are highlighted in orange (if detected in this study) or in green (if not detected in this study).

To further explore the function of Rac1 in retina, the mouse Rac1 interactome was constructed in InnateDB^31^ (Fig. 5); 133 interactions were mapped. Expression profiles were added to the network from Supplementary Table S1^11^,^23^, which indicated that 114 (86%) of these proteins were expressed in retina. Expression of 29 members (~25%) was detected in our LC-MS/MS analysis (Fig. 5, Supplementary Table S5a). Of the direct interactors of Rac1, Rap1gds1 and Cav1 were significantly upregulated (~2-fold, p < 0.01), while Nckap1 was down-regulated (~50%, p < 0.01) in R347 versus wt retinas (Fig. 5, Supplementary Table S5a). Many of the detected proteins clustered in the IDB-8002 complex^32^ (Fig. 5). These proteins connect to the Rac1 network via Mtnr1a, which is expressed at a very low level in retina (0.43 FPKM/RPKM; Supplementary Table S1). A number of IDB-8002 proteins were significantly up-regulated in R347 versus wt retinas including Rab10, Pgrmc1 and Pdia6 (Fig. 5; Supplementary Table S5a) while others, e.g. Gnb1, were down-regulated (Fig. 5; Supplementary Table S5a). Rac1 interactions with 22 proteins in photoreceptor outer segments have been identified^33^. We added the mouse orthologs of these proteins to the interactome (Fig. 5; Supplementary Table S5b). Nine of the 22 proteins were detected and analysed in our LC-MS/MS dataset (Fig. 5, Supplementary Table S5b). Expression of Eno1 and Sag was decreased (Fig. 5, Supplementary Table S5b) while expression of other proteins, such as Prdx2 and RhoA, did not change significantly in R347 versus wt retinas (Fig. 5; Supplementary Table S5b).

## Discussion

Previously, six miRNAs with altered expression in R347 mouse retinas were identified^12^. In this study, a proteome map of the R347 model was generated using high-throughput LC-MS/MS (Fig. 1). Proteomics data was combined with *in silico* target predictions for these six miRNAs to identify potential miRNA-target mRNA pairs. Validation of candidate miRNA-target mRNA interactions was undertaken and possible associations between identified miR regulatory circuits and cellular function(s) explored.

As the retina is a complex tissue composed of many cell types, in some cases, it is possible that the determined miRNA^12^ and protein changes may have occurred independently in different retinal cell types. Additionally, changes of protein and miRNA levels in R347 versus wild type retinas may have resulted from changes in retinal cell composition due to the progressive retinal degeneration in R347 mice. To minimise potential misinterpretation of data due to the above, cellular colocalisation of the three targets followed up (i.e. Rac1, Ctbp2 and Slc6a9) and their targeting miRNAs was shown (Fig. 3 and Table 2). For example, both miR-183/96/182^12^,^15^ and Rac1 have a pan-retinal expression pattern, while miR-1 and Ctbp2 are coexpressed in photoreceptor cells (Fig. 3 and Table 2). To minimise changes in cell composition due to degeneration in R347 retinas, one-month old animals were used. By this age, maturation of the retina is complete, while photoreceptor cell loss is still relatively modest, i.e. approximately 25% 12,18. Additionally, when analyzing miRNA and protein changes we focused on alterations in excess of ± 25% (i.e. < 75% or > 125% of wt levels). For example, in pathway over-representation analysis, we set cut-off values of <0.5-fold or >2-fold change (p < 0.05) in individual protein levels between R347 versus wt samples. Notably, while controlling error, we used the miRNA and corresponding protein changes only to predict potential miRNA-target mRNA interactions. For three selected targets (i.e. Rac1, Ctbp2 and Slc6a9) interactions were shown by *in vitro* UTR assays. For Rac1, miR-183/96/182 interactions were additionally demonstrated using *in vivo* miR-CATCH.

As the retina is rich in membranes and associated proteins, beside standard protein extraction from whole retinas, proteins were extracted from enriched retinal membranes^34^, thereby increasing number of identified protein IDs by ~82% (Fig. 1b). We estimated that our quantitative LC-MS/MS enabled analysis of ~9.7% of the retinal proteome, indicating superior coverage compared to prior studies of mouse retina, e.g.^35^.

811 proteins exhibited differential expression between R347 and wt mouse retinas. To explore the possible function(s) of these proteins in retina, pathway over-representation analysis was performed^24^. Most up-regulated pathways in R347 versus wt retinas were related to signal transduction and synaptic plasticity (Fig. 2a). A number of up-regulated pathways were associated with semaphorin interactions (Fig. 2a), secreted transmembrane proteins involved in nervous system development, axon guidance, neuronal plasticity and degeneration^36^. Synaptic plasticity and reorganisation may represent functions of these in the R347 retina. For example, the observed alteration in Ctbp2 expression (Fig. 3c,d) may reflect synaptic remodeling in photoreceptors^37^. Semaphorins also regulate the cytoskeleton, cell adhesion, and survival; such functions could also be targeted in degenerating retinas. Another key group of up-regulated pathways involved TGF-beta receptor and TNF-alpha/NF-kB cytokine signaling (Fig. 2a). TGF-beta is involved in regulation of cell proliferation and differentiation, and is known to influence microglia activation^38^ and developmental apoptosis in retina^39^. Microglia activation and apoptosis characterize the R347 retina; the TGF-beta receptor may play a role in these processes. TNF-alpha can be pro- or anti-apoptotic depending on the pathway involved. However, it may have an adverse role in retina as TNF-alpha blockers suppressed retinal damage in a retinal ischemia model^40^. Most down-regulated pathways in R347 retina were related to phototransduction (Fig. 2a). As photoreceptor outer segments, the subcellular compartment for phototransduction, are compromised in R347 retinas, it is likely that decreased levels of some of these proteins were due to loss of outer segments rather than actual reduction in expression level.

Unless noted, according to miRTarBase^41^, the miRNA-target mRNA interactions reported here have not been described previously. miR-1 and miR-133 form a miRNA cluster and can influence neuronal function^42^. We previously reported a marked up-regulation of miR-1/133 in mouse models of RP^12^. Our data suggest that miR-1 may target three functional axes in the R347 retina; G-protein signaling/visual transduction, mitochondrial function, and synaptic transmission (Fig. 2b, Supplementary Table S3). We validated miR-1 targeting of Ctbp2 in a 3′UTR assay (Fig. 3h); notably, the human equivalent miRNA (hsa-miR-1-3p) has also been shown to target CTBP2 in HeLa cells^43^. As the Ctbp2 3′UTR also has a predicted target site for miR-133, miR-1/133 may co-target Ctbp2 (Fig. 3g); however this was not tested in our study. The miR-1/133 cluster and Ctbp2 are co-expressed in photoreceptors; expression of both miR-1 and miR-133 is increased by ~20-fold in R347 versus wt photoreceptors (Table 2)^12^. Ctbp2 levels were reduced by ~50% (LC-MS/MS) and Ctbp2 immunolabeling was reduced in photoreceptor synaptic regions (Fig. 3c,d) in R347 retinas; a similar reduction of Ctbp2 has been observed in synaptic remodeling following retinal detachment^37^. The data above suggest that miR-1 suppresses Ctbp2 in R347 retinas and that miR-1 (and possibly miR-133) may regulate synaptic remodeling at photoreceptor synapses by targeting Ctbp2.

The sensory organ-specific miR-183/96/182 cluster has been studied extensively in retina^15^; e.g., its inactivation results in progressive RD in mice^16^. Remarkably, expression of miR-183/182 is sufficient to maintain outer segments and expression of cone opsins in cone photoreceptors^17^. Notably, the miR-183/96/182 cluster is regulated by light; it is down-regulated in dark-adapted and up-regulated in light-adapted retinas^8^. Pathways potentially regulated by miR-183 include GABA receptor activation, L1cam-mediated interactions and L1 signal transduction (Fig. 2b). A significant overlap between miR-96 and miR-182 targets was established involving enriched pathways for solute carrier-mediated transmembrane transport and Robo receptor signaling. Pathways potentially regulated by miR-96 exclusively included execution of apoptosis and integrin mediated cell adhesion (Fig. 2b); whereas those identified for miR-182 comprised transmembrane transport of small molecules, G-protein signaling, synaptic transmission and cell-adhesion (Fig. 2b). Atp1b3, Paip2b and Slc1a1 have been previously identified as retinal targets for miR-183/96/182^8^. Our data further validates Atp1b3 and possibly Slc1a1 targeting by this miR cluster in R347 retina as their expression increased by 31.6% (p < 0.001) and 38.9% (p > 0.05), respectively; Paip2b was not detected.

One of the miR-96/182 targets identified in this study was Slc6a9; these miRNAs and Slc6a9 are co-expressed in photoreceptors (Fig. 3e,f and Table 2)^12^,^17^,^44^. *In vitro* 3′UTR assays confirmed miR-96 targeting of Slc6a9, while miR-182 (a target site with lower conservation) did not suppress Slc6a9 (Fig. 3h). Additionally, we have demonstrated that Slc6a9 is exclusively expressed in cone photoreceptors (Supplementary Figure S2). While ‘uneven’ expression of Slc6a9 in the outer nuclear layer was previously observed^44^, to our knowledge, cone-specific expression of Slc6a9 has not been demonstrated before.

Another interesting target identified for the miR-183/96/182 cluster was Rac1 (Fig. 3a,b and Table 2). Rac1 is an essential component in the CNS where it regulates axon growth, neuronal morphology and survival^45^. In photoreceptor cells, a key subcellular compartment for Rac1 is the outer segment^46^,^47^. Here, Rac1 is activated by intense light via binding to rhodopsin^45^,^47^. In a light-induced photoreceptor degeneration model, Rac1 was activated while its mRNA expression also increased^22^. Rac1 activation was also demonstrated in a diabetic retinopathy model^21^. This may be due to Rac1′s involvement as a component of the NADPH oxidase system^48^, which contributes, for example, to diabetes-induced oxidative stress in the retina^49^. In the present study, we established more than 3-fold increase in Rac1 protein level in whole retina protein extracts in R347 versus wt mouse retinas. We also found a less prominent decrease (~40%) of Rac1 in the retinal membrane protein extracts; we speculated that this reduction in membrane bound Rac1 level could have been caused by either protein relocation and/or the marked loss of rod outer segments (a major membrane component) in R347 retinas, rather than *bona fide* alteration in Rac1 expression. Other studies also suggest that up-regulation of Rac1 could be a common feature in photoreceptor degenerations. For example, constitutive activation of Rac1 in developing rods results in rod mislocalization, lack of formation of segments, and abnormal synaptic localization^20^. Conditional knockdown of Rac1 in photoreceptors provided protection against light-induced photoreceptor death and did not have negative effects on retinal structure and function^26^. As Rac1 is a component of the NADPH oxidase system that produces reactive oxygen species^48^, protection in this model may relate to modulation of this system.

To learn about potential Rac1 regulatory circuits in retina, the Rac1 interactome was constructed and interrogated (Fig. 5 and Supplementary Table S5). We detected ~25% of the interactome members by LC-MS/MS; of the direct interactors, Rap1gds1 and Cav1 were significantly up-regulated (~2-fold, p < 0.01) in R347 retinas. Rap1gds1 is a stimulatory GDP/GTP exchange protein, which activates RhoA and Rac1^50^. Parallel up-regulation of Rap1gds1 and Rac1 therefore suggests a marked activation of Rac1. Cav1 is a key component of caveolae plasma membranes where it interacts with various signaling molecules^51^. Cav1 is primarily expressed in retinal Muller cells (Supplementary Figure S1)^52^ suggesting activation of the Rac1 axis in glial cells. Additionally, nine Rac1 photoreceptor outer segment interactors (reported previously)^33^ were detected in our LC-MS/MS study (Fig. 5 and Supplementary Table S5b). Levels of these proteins either decreased (such as Eno1 and Sag) or did not change significantly (e.g. Prdx2 and RhoA). Interpretation of these data was hampered by the significant loss of outer segments in R347 retina, which likely interfered with these protein levels.

Rac1 and miR-183/96/182 are co-expressed in retinal cells (Fig. 3a,b and Table 2)^12^. miR-96/182 targeting of Rac1 was validated for both miRNAs by *in vitro* 3′UTR assays (Fig. 3g,h). Additionally, using miR-CATCH (Fig. 4)^27^, we established that both miR-96 and miR-182 interact with Rac1 *in vivo* in retina. Note, that the Rac1 3′UTR also contains a predicted miR-142 target site, functionality of which we did not test. As hsa-miR-142 targeting of RAC1 was shown in human hepatocellular carcinoma cell lines^53^, miR-142 targeting of Rac1 is also possible. Our data suggest that miR-183/96/182 cluster has a significant influence on Rac1 expression and that this regulatory circuit may play an important role in both healthy and RP retinas. Linking our results to current knowledge suggests that in retina, parallel to activation of Rac1 46, light up-regulates expression of the miR-183/96/182 cluster^8^, which in turn may provide negative feedback to Rac1 translation. Additionally, reduced expression of miR-183/96/182 cluster in R347 retinas may decrease efficacy of this feedback mechanism and contribute to elevated Rac1 levels.

While the miR-183/96/182 cluster has an essential role in sensory organs, these miRNAs are also implicated in regulation of non-sensory cells and disorders (e.g. cancer)^54^. Previously established functions for miR-183/96/182 include regulation of circadian rhythm, apoptosis^54^, photoreceptor differentiation and synaptic connectivity^15^. Our data confirm apoptosis, signal and synaptic transduction and add two novel categories; transmembrane transport and cell-adhesion (Fig. 2b, Supplementary Table S3). Based on combination of *in silico* target computation and high throughput proteome analysis, we predicted more than a hundred miRNA-target mRNA interactions in retina (Supplementary Table S3). We tested five of these interactions using miR-CATCH and/or 3′UTR assays and validated four interactions suggesting that our predictions are robust. As such, many other genes in Supplementary Table S3 may also represent genuine targets for these miRNAs, which could be validated in further studies.

A combination of *in silico* miRNA target predictions and Rac1-miR-CATCH data was explored to identify additional miRNAs, which may target Rac1 mRNA in retina. Apart from the conserved miR-96/182 target site, eight additional miR-183/96/182 target sites in Rac1 were predicted (Fig. 4d and Table 3). Beside miR-183/96/182, a number of other miRNAs were both predicted to target the Rac1 3′UTR and were significantly enriched in Rac1-miR-CATCH. These included miR-103-3p, let-7a/c/e/f-5p, miR-320-3p, miR-101a-3p and miR-672-5p (Fig. 4d and Table 3). Reviewing miRTarBase^41^, human miRNAs, i.e. hsa-miR-101 and hsa-miR-320a targeting of RAC1 had been observed in cells^55^,^56^, supporting the current findings with these miRNAs in mouse retina.

While widespread binding of miRNAs to mRNA coding regions has been documented^4^,^55^,^57^, most prediction tools focus on 3′UTRs. Notably, miR-CATCH detects any mRNA-bound miRNAs independent of the location of the site^27^. RNA22^30^ analysis of the Rac1 cDNA was used to search for miRNA target sites outside the 3′UTR. Target sites in the 5′UTR and coding region, which were both *in silico* predicted and the corresponding miRNAs enriched in Rac1-miR-CATCH comprised let-7a/e-5p, miR-9-5p, miR-26a-5p, miR-151-3p and miR-652-3p (Fig. 4d and Table 3a). Two of these miRNAs, i.e. mmu-miR-124-3p^58^ and hsa-miR-652-3p^59^, have been shown to target Rac1/RAC1, respectively. Other Rac1-miR-CATCH-captured miRNAs, which were not predicted to target Rac1 included miR-125a/b-5p, miR-378a-3p and miR-204-5p; all of which had ≥4-fold enrichment (Table 3b). Non-canonical modes of miRNA binding^60^, not identified by miRNA target site prediction algorithms, may underlie the enrichment for these miRNAs. Our data suggest that more than 30 miRNAs may interact with Rac1 mRNA in retina, targeting the 5′UTR, coding region and 3′UTR, using both canonical and non-canonical modes of action (Fig. 4d and Table 3).

miRNA dysregulation is a hallmark of RD. Focusing on predicted targets for modulated miRNAs in R347 retina, including miR-1/133, miR-142 and miR-183/96/182, high-throughput proteome analysis provided a unique opportunity to explore miRNA regulation in a model system for inherited retinopathy. Our results highlight widespread effects of these miRNAs in retina, in particular, miR-1 and the miR-183/96/182 cluster; we validated a number of specific miRNA-target interactions *in vitro* and *in vivo*. Key cellular functions identified as being regulated by these miRNAs include signal transduction, synaptic transmission, cell-adhesion and transmembrane transport. Exploiting combination of miR-CATCH and *in silico* miRNA target predictions, we propose extensive miRNA-Rac1 mRNA interactions in retina, including targeting both coding and non-coding regions. A number of Rac1-miR-CATCH-enriched miRNAs not predicted *in silico*, suggest that non-canonical miRNA targeting of Rac1 may be common. The high-throughput proteome analysis enabled quantitative evaluation of expression of ~25% of the retinal Rac1 interactome. Significant utilisation of miRNA-based regulation, a number of miRNA targets, and possible miRNA-regulated cellular pathways and functions were identified in retina; some of these interactions may represent potential targets for future therapeutic intervention for RD.

## Methods

### Animals

Pro347Ser rhodopsin (RHO-Pro347Ser) transgenic mice (on 129 background)^18^ were crossed to rhodopsin+/− mice (Rho+/−; also on 129 background)^19^, giving RHO-Pro347Ser+/−Rho+/−genotype (R347); wild-type 129 mice (wt) were used as controls. Mice were maintained under specific pathogen free (SPF) housing conditions and analysed at one month of age. Animal experiments were carried out in accordance with the Directive 2010/63/EU; Protection of Animals Used for Scientific Purposes, Regulations 2012 [S.I. No. 543 of 2012]. The experimental protocols were approved by Trinity College Dublin Animal Research Ethics Committee (AREC) and authorized by the Irish Medicines Board (IMB).

### *In silico* miRNA Target Selection Pipeline

Target sites for mmu-miR-1a-3p (miR-1), miR-133a-1 (miR-133), miR-142a-3p (miR-142), miR-183-5p (miR-183), miR-96-5p (miR-96) and miR-182-5p (miR-182) were predicted employing Diana-microT (v.3.0)^61^, miRanda (Aug 2010 release)^62^ and TargetScan tools (v.6.2)^4^, and filtered for sites predicted by at least two prediction tools. Other databases used included miRSystem (version Mar 2015)^29^, RNA22 (version 2)^30^ and miRTarBase (Release 6, Sept. 2015)^41^. A wt mouse retinal transcriptome library was constructed using RNA-seq data^11^,^23^. Expression levels were determined in FPKM (fragments per kb of transcript per million reads)^23^ and in RPKM (reads per kb of transcript per million reads)^11^. While FPKM and RPKM are slightly different definitions, importantly both of them are normalized values and in effect have the same meaning. For the purposes of the current analysis, they were treated as interchangeable.

### Proteome Analysis

LC-MS-MS and label-free quantification were performed as described^54^. LC-MS/MS analysis was carried out on an Ultimate3000 nano HPLC system (Dionex, Sunnyvale, CA, USA) coupled to a LTQ OrbitrapXL mass spectrometer (Thermo Fisher Scientific Inc., Waltham, MA, USA)^34^. MS spectra were acquired in OrbitrapXL and up to 10 of the most abundant peptide ions selected for fragmentation in the linear ion trap. Peptides were quantified using Progenesis QI (Waters, Milford, MA, USA) and identified with Mascot (version 2.5; Matrix Science, Boston, MA, USA) software. Statistical significance was determined using ANOVA and p < 0.05 values were regarded as statistically significant. Gene Ontology (GO, http://geneontology.org) analysis was carried out using ‘membrane’ as query term. Pathway enrichment analysis in ConsensusPathDB (Release MM9)^24^ was performed using Reactome (http://www.reactome.org), Wikipathways (http://www.wikipathways.org) and MouseCyc (http://mousecyc.jax.org) databases; the minimal overlap with the input list and the p value cut off were set to 2 and 0.05, respectively. The mouse Rac1 interactome was generated in InnateDB^31^. Subcellular localization was confirmed using Uniprot (http://www.uniprot.org) and GeneCards (http://www.genecards.org), and modified for a number of genes, in particular for the ones with ‘unknown’ localization. Mouse orthologs of previously identified photoreceptor outer segment Rac1 interactors were added manually^33^.

### Immunohistochemistry

Mouse eyes were fixed in either 4% paraformaldehyde or 4% paraformaldehyde/2% acetic acid at 4 °C for 4 h. Eyes were washed, cryoprotected, embedded in PolyFreeze (Sigma-Aldrich, Arklow, Ireland). 12 μm cryosections were processed for Api5 (ProteinTech, Manchester, UK; 1:300 dilution), Arcn1 (SantaCruz Biotechnology Inc, Heidelberg, Germany; 1:100 dilution), Cav1 (Pierce Antibody Products, Thermo Fisher Scientific; 1:100 dilution), Ctbp2 (BDUK, Oxford, UK; 1:300 dilution), Dgke (ProteinTech; 1:100 dilution), Flot2 (Pierce Antibody Products; 1:200 dilution), Igf1r (Cell Signaling Technology, Danvers, MA, USA; 1:100 dilution), Negr1 (SantaCruz Biotechnology Inc; 1:100 dilution), Rac1 (Millipore Ireland B.V. Carrigtwohill, Ireland; 1:300 dilution), Slc6a9 (Santa Cruz Biotechnology Inc.; 1/100 dilution), Tagln3 (ProteinTech; 1:100 dilution) immunohistochemistries using overnight incubation at 4 °C. Some sections were co-incubated with 20 μg/ml of lectin PNA-AlexaFluor-488 conjugate (Molecular Probes, Thermo Fisher Scientific). Sections were then incubated with Cy3-conjugated secondary antibodies (Jackson ImmunoResearch Laboratories, Newmarket, UK; 1:400 dilution) for 2 h and counterstained with DAPI. Specimens were analysed with an Axiophot fluorescent microscope (Zeiss UK, Cambridge, UK) and images acquired utilising AnalySIS^B software (Soft Imaging System, Muenster, Germany). Images taken with different filter sets were overlaid in Adobe Photoshop CS6 (Adobe Systems Software Ireland Ltd, Dublin).

### *In Vitro* 3′UTR Assay

Mouse Rac1 (NM_009007), Ctbp2 (NM_009980) and Slc6a9 (NM_008135) 3′UTR sequences in dual firefly luciferase/Renilla luciferase expression vectors (MmiT028031, MmiT028960 and MmiT027208) were obtained from GeneCopoeia Inc. (Rockville, MD, USA). Synthetic pre-miRNAs for mmu-miR-1a-3p (PM10617), mmu-miR-96-5p (PM10422), mmu-miR-182-5p (PM13088) and negative control were procured from Ambion (Thermo Fisher Scientific). 1.4 × 10^5^ HeLa cells/well were seeded in 24-well plates; 24 h later, cells were co-transfected with 400 ng 3′UTR plasmids and 16 pmoles of each pre-miRNAs using Lipofectamine 2000 (Invitrogen, Thermo Fisher Scientific). 24 h post-transfection, cells were analysed using a Dual-Glow luciferase assay system (Promega, Madison, WI, USA). ANOVA and post-hoc Tukey’s multiple comparison test were performed and p < 0.05 values were accepted as statistically significant.

### *In Vivo* miRNA Capture Affinity Technology (miR-CATCH)

To co-purify Rac1 mRNA with its endogenously bound miRNAs, miRNA capture affinity technology (miR-CATCH)^27^ was adapted for mouse retina. Two 3′-biotinylated capture oligonucleotides were designed with M-Fold^63^ for regions predicted to be single-stranded at 37 °C in 500 mM salt solution (C9: 5′-AGGAAATGCATTGGTCGTGTAA-3′; C10: 5′-GATGATAGGAGTATTGGGAC-3′). Complementarity to off-targets was determined using Nucleotide Blast^28^ and set to ≤15 bp. A 3′-biotinylated non-targeting scrambled control oligonucleotide (5′-GTGAGGCGTTGTAAGAGTGGTTAAG-3′) was designed similarly. Mouse retinas were trypsin dissociated in 1.5 ml HBSS^64^. Cells were fixed in 0.2% formaldehyde 10 min, washed in 0.2 M glycine, then in PBS and pelleted. Cell lysis was performed in 0.7 ml FA lysis buffer (50 mM HEPES pH 7.5, 140 mM NaCl, 1 mM EDTA, 1% Triton, 0.1% sodium deoxycholate) supplemented with a protease inhibitor cocktail (Roche Diagnostics Limited, Burgess Hill, UK), 6 μM PMSF and 120 U RNasin (Promega) using 0.5 mm glass beads and a FastPrep cell disrupter (MP Biomedicals, Santa Ana, CA, USA). 20 mM EDTA was added to the cell lysate and cell debris removed by centrifugation. 0.8 nmoles of capture and scrambled oligonucleotides were separately immobilised on 200 μl MyOne Streptavidin C1 magnetic beads (Life Technologies, Thermo Fisher Scientific). Oligonucleotide-complexed beads were resuspended in 0.7 ml hybridisation buffer (2X TE buffer, pH 7.5, 1 M LiCl, 37 °C) and 0.5 ml 1X Tris-EDTA buffer (37 °C). 200 μl of cell lysate was added and samples incubated at 37 °C for 2 h. Beads were washed four times with buffer A (1X TE buffer, pH 7.5, 0.15 M LiCl, 0.5% SDS) and once with buffer B (1X TE buffer, pH 7.5, 0.15 M LiCl). RNA was eluted by incubating the beads in 1 mg/ml proteinase K solution (in TE, 0.1 M NaCl, 0.5% SDS) for 1 h at 37 °C with agitation and for 10 min at 95 °C.

### RNA Isolation, mRNA and miRNA Expression Analysis

Total RNA from retina and miR-CATCH samples was isolated using the miRCURY Cell & Plant kit (Exiqon A/S, Vedbaek, Denmark). An on-column DNase step (1 h at 37 °C) was added to the protocol using RNase-free DNase (Qiagen Ltd., Manchester, UK). RT-qPCR was performed for Rac1 (NM_009007.2), Folr1 (XM_006507360.1), Plxna4 (NM_175750.3), Tmed5 (XM_006535240.1), Ttc21b (NM_001047604.1) and Actb (NM_007393.3) mRNAs; primer sequences are given in Supplementary Table S6. RT-qPCRs were performed in triplicates using QuantiTect SYBR Green RT-PCR Kit (Qiagen) in a StepOnePlus real-time PCR system (Applied Biosystems, Thermo Fisher Scientific); Actb was used for normalisation. Typical dilutions of RNA samples in the final PCR reaction mixture were 1/200 and 1/1200 for miR-CATCH and retinal RNAs, respectively.

miRNA expression was analysed at Exiqon Services (Vedbaek, Denmark) and in-house. Exiqon miRNA PCR panel profiling (372 miRNA assays) was performed according to the company’s assay pipeline. RNA was reverse transcribed using the miRCURY LNA Universal RT microRNA PCR, Polyadenylation and cDNA synthesis kit (Exiqon). cDNA was assayed using the protocol for miRCURY LNA Universal RT microRNA PCR. Each miRNA was assayed once on the microRNA Ready-to-Use PCR, Mouse & Rat panel I (Exiqon rodent miRNA PCR panel) using ExiLENT SYBR Green master mix and LightCycler 480 Real-Time PCR System (Roche Diagnostics, Indianapolis, IN, USA). As the miR-CATCH capture and scrambled control samples were expected to have different RNA compositions, no normaliser was used and comparison of the background-filtered raw expression data was performed. For in-house TaqMan microRNA assays (Applied Biosystems) 5 μl of miR-CATCH capture and scrambled RNA sample was reverse-transcribed using TaqMan MicroRNA Reverse Transcription Kit (Applied Biosystems). One μl of the RT reaction was analysed in triplicate in StepOnePlus real-time PCR system (Applied Biosystems) using TaqMan Universal Master Mix II (Applied Biosystems). Quantification was performed utilising the comparative Ct method^65^. Statistical analysis was performed using Student’s t-Test and differences with p < 0.05 values were accepted as statistically significant.

## Additional Information

**How to cite this article**: Palfi, A. *et al*. microRNA regulatory circuits in a mouse model of inherited retinal degeneration. *Sci. Rep.* **6**, 31431; doi: 10.1038/srep31431 (2016).

## Supplementary Material

Supplementary Information

Supplementary Table 1

Supplementary Table 2

Supplementary Table 3

Supplementary Table 4

## Figures and Tables

**Table 1:** *In silico* miRNA target prediction pipeline.

	Prediction method			Filter		
miRNA	microT^61^	miRanda^62^	Target-Scan^4^	Predicted by ≥2 methods	Overlaping predicted target sites	Expressed in retinal transcriptome library^11^,^23^
miR-1	7081	2493	2378	1264	985	885
miR-133	5795	1611	1946	912	696	606
miR-142	3753	1834	1371	711	555	478
miR-183	1235	2399	1911	840	567	523
miR-96	9844	2216	2451	1472	1136	1031
miR-182	11484	2850	2563	1711	1362	1195
Sum	39192	13403	12620	6910	**5301**	**4718**
Unique	25556	8416	6917	4574	**3721**	**3262**

The number of candidate genes for each miRNA, the sum of candidate genes for the six miRNAs (Sum) and the number of non-redundant candidates for the six miRNAs (Unique) are given.

**Table 2:** Selected miRNA and candidate target protein levels in R347 versus wt retinas.

Target mRNA/gene	Target protein level in R347 versus wt whole retinal protein extract (LC-MS/MS)	Target protein level in R347 versus wt retinal membrane protein extract (LC-MS/MS)	Predicted targeting miRNA	miRNA expression in R347 versus wt retina	miRNA expression in R347 versus wt photoreceptor
Rac1	339.9 ± 55.9%(p < 0.001)	60.3 ± 9.8%(p < 0.05)	miR-96	~50%	~60%
			miR-182	~50%	~60%
			miR-142	~400%	~1000%
Ctbp2	53.8 ± 13.6%(p < 0.01)	not detected	miR-1	~550%	~2000%
			miR-133	~500%	~2000%
Slc6a9	174.2 ± 14.1%(p < 0.05)	167.5 ± 11.2%(p < 0.001)	miR-96	~50%	~60%
			miR-182	~50%	~60%

miRNA targets were predicted using an *in silico* prediction pipeline employing microT 61, miRanda^62^ and TargerScan^4^ tools. Target protein levels were determined using label-free LC-MS/MS in R347 and wt retinas. miRNA levels were taken from^12^.

**Table 3:** miRNA targeting of Rac1.

miRNA	miR-CATCHenrichment	p value	Number of targetsites	Targetregion	Reference
a					
miR-103-3p	9.54	0.0012	1	3′	
let-7e-5p	5.53	0.1649	2	C, 3′	
miR-9-5p	5.37	0.0381	3	C	
miR-96-5p	5.07	0.0063	3	3′	
miR-26a-5p	4.74	0.1366	1	C	
let-7c-5p	4.35	0.0459	4	5′, 3′	
miR-16-5p	4.03	0.0802	1	C	
miR-320-3p	3.91	0.0906	5	5′, 3′	56
miR-484	3.68	0.1207	1	3′	
miR-182-5p	3.63	0.0158	5	C	
miR-24-3p	3.31	0.0695	2	5′, C	
miR-101a-3p	3.23	n/a	1	3′	55
miR-181a-5p	2.99	0.0279	2	3′	
miR-99a-5p	2.90	n/a	2	3′	
miR-124-3p	2.82	0.0484	1	C	56
miR-183-5p	2.53	0.0327	2	C, 3′	
miR-184-3p	2.24	0.0981	3	C, 3′	
miR-107-3p	2.15	n/a	1	3′	
let-7a-5p	1.65	0.0957	3	5′, 3′	
let-7f-5p	n/a	n/a	1	3′	
miR-151-3p	n/a	n/a	2	C	
miR-652-3p	n/a	n/a	1	C	59
miR-672-5p	n/a	n/a	5	5′, C, 3′	
miR-139-5p	n/a	n/a	1	3′	
b					
miR-125a-5p	7.38	n/a	n/a	n/a	
miR-125b-5p	5.18	0.0822	n/a	n/a	
miR-378a-3p	4.14	n/a	n/a	n/a	
miR-204-5p	3.95	n/a	n/a	n/a	
miR-181b-5p	3.80	0.0229	n/a	n/a	
miR-30c-5p	3.10	0.0174	n/a	n/a	59
miR-328-3p	2.79	0.1867	n/a	n/a	
miR-211-5p	2.76	0.1614	n/a	n/a	
miR-3107-5p	2.38	n/a	n/a	n/a	

miRNAs targeting Rac1 were predicted *in silico* using miRSystem^29^ and RNA22^30^, and compared to miRNAs enriched via *in vivo* retinal Rac1-miR-CATCH. The miRNAs from the intersection of these two lists are given in part **a**, while miRNAs enriched in Rac1-miR-CATCH but not predicted *in silico* to target Rac1 are given in part **b**. miRNAs are listed in order of miR-CATCH enrichment value and corresponding p values (Student’s t-Test) are provided. Note, that p values were not calculated if one or both of the scrambled control samples were not amplified (n/a); enrichment values were not calculated if the scrambled control samples were not amplified (n/a). The number of predicted target sites, the location of the predicted target sites (5′: 5′UTR, C: coding sequence, 3′: 3′UTR) and reference if targeting has previously been reported are given.
