# Supplementary material for: microRNA regulatory circuits in a mouse model of inherited retinal degeneration
Source: Sci Rep. 2016 Aug 16;6:31431. doi: 10.1038/srep31431 (PMC4985623; doi:10.1038/srep31431)
Supplement: Supplementary Information [file srep31431-s1.doc]

**Supplementary Material**

**microRNA regulatory circuits in a mouse model of inherited retinal degeneration**

Arpad Palfi, Karsten Hokamp, Stefanie M. Hauck, Sebastian Vencken, Sophia Millington-Ward, Naomi Chadderton, Mathew Carrigan, Elod Kortvely, Catherine M. Greene, Paul F. Kenna and G. Jane Farrar.

**Supplementary Table S1** Wild type mouse retinal transcriptome library. Two RNA-Seq datasets [11, 23] were combined. Genes with an expression value over 0.5 FPKM/RPKM in at least one of the sources were deemed as being expressed in retina resulting in 14335 out of 22788 expressed genes (separate Excel file).

**Supplementary Table S2** LC-MS/MS analysis of retinal protein expression between wt and R347 mice. Protein levels were determined from whole retina protein (n=4) and retinal membrane protein (n=4) extracts using label-free LC-MS/MS. Accession numbers, corresponding peptide counts, confidence scores, p values (ANOVA) and wt versus R347 ratios of the identified proteins are given (separate Excel file).

**Supplementary Table S3** Putative retinal target genes for miR-1, miR-133, miR-142, miR-183, miR-96 and miR-182. Data from *in silico* miRNA target predictions (5301 target genes) and retinal proteome analysis (1446 genes) were combined. Proteins with significantly altered expression (p<0.05) and with inverse relation to changes in levels of predicted targeting miRNAs in R347 versus wt retinas were selected. 133 target genes meeting these criteria in either the whole retina protein extract and/or the retinal membrane protein extract are listed by targeting miRNAs. 23, 10, 6, 18, 35 and 41 potential target genes were identified for miR-1, miR-133, miR-142, miR-183, miR-96 and miR-182, respectively. MGI gene symbols, names, descriptions, associated diseases, corresponding protein IDs, wt versus R347 ratios of protein levels (LC-MS/MS), p values (ANOVA; LC-MS/MS), peptide counts (LC-MS/MS), confidence scores (LC-MS/MS), target prediction methods (D: Diana-microT [61]; M: miRanda [62]; T: TargetScan [4] are given for each target (separate Excel file).

**Supplementary Figure S1**

**
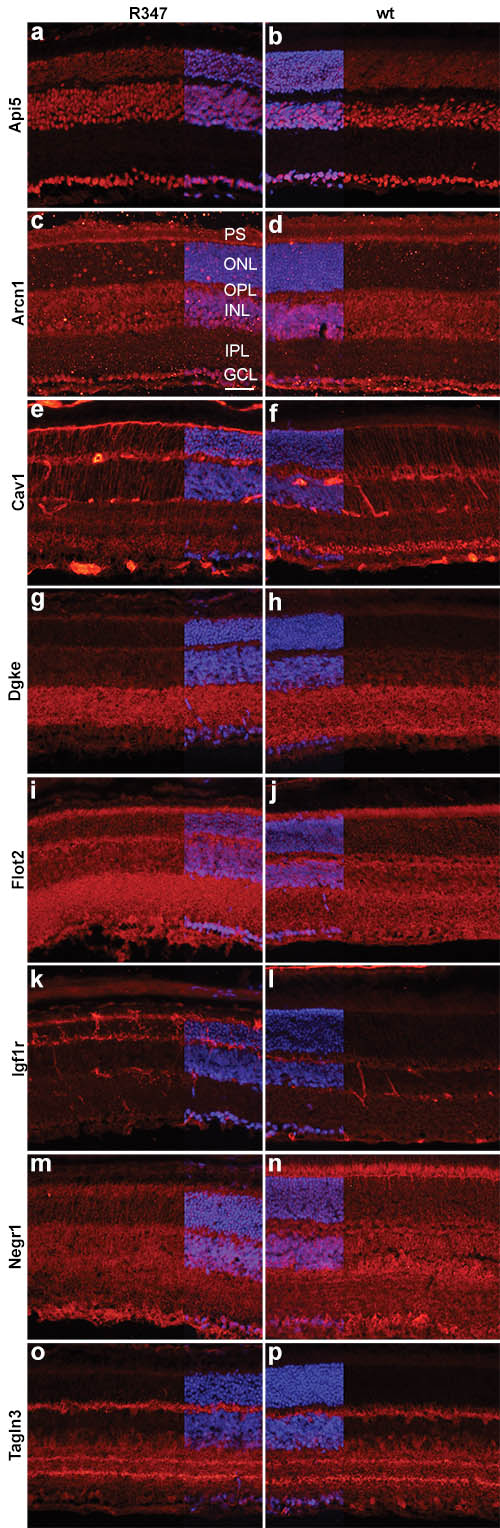
**

**Supplementary Figure S1** Retinal expression of potential miRNA target proteins in R347 and wt mice. Cy3-labeled immunohistochemistry was performed for Api5 (**a** and **b**), Arcn1 (**c** and **d**), Cav1 (**e** and **f**), Dgke (**g** and **h**), Flot2 (**i** and **j**), Igf1r (**k** and **l**), Negr1 (**m** and **n**) and Tagln3 (**o** and **p**) in retinal cryosections (12 μm) of R347 and wt mice at one month of age (n=3). DAPI was used for nuclear counterstaining; DAPI signals were overlaid only on 1/3 of the images to enable better visualization of the Cy3 label. Scale bar represents 25 μm (panel c). GCL: ganglion cell layer, INL: inner nuclear layer, IPL: inner plexiform layer, ONL: outer nuclear layer, OPL: outer plexiform layer, PS: photoreceptor segment layer.

**Supplementary Figure S2**


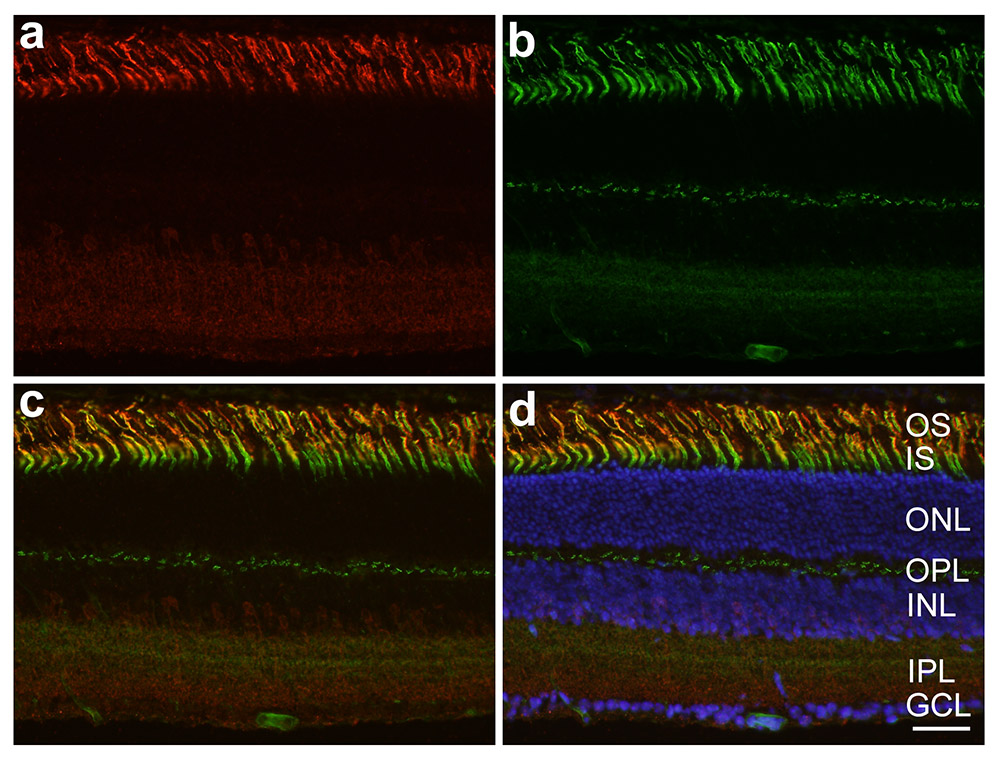


**Supplementary Figure S2** Slc6a9 and lectin PNA co-labeling in mouse retina. Slc6a9 immunohistochemistry (Cy3 detection) and lectin PNA-AlexaFluor-488 conjugate labeling was performed in retinal cryosections (12 μm; n=3) from one month-old wt mice. DAPI was used for nuclear counterstaining. **a** Slc6a9 (Cy3) signal; **b** PNA-AlexaFluor-488 conjugate signal; **c** Slc6a9 (Cy3) and PNA-AlexaFluor-488 conjugate signals overlaid; **d** Slc6a9 (Cy3), PNA-AlexaFluor-488 conjugate and DAPI signals overlaid. Scale bar represents 25 μm (panel d). GCL: ganglion cell layer, INL: inner nuclear layer, IPL: inner plexiform layer, IS: photoreceptor inner segment layer, ONL: outer nuclear layer, OPL: outer plexiform layer, OS: photoreceptor outer segment layer.

**Supplementary Table S4** Exiqon rodent miRNA PCR panel expression profiling of Rac1-miR-CATCH samples. Fold enrichment and p values (Student’s t-Test) in capture versus scrambled control samples were calculated (n=2). ND: not detected. p values were not calculated if the corresponding miRNAs were not detected in the control samples (separate Excel file).

**Supplementary Table S5**

**a**

| Gene | Expression level (log2 of  FPKM/RPKM) | wt/R347 protein level in whole retina protein extract | wt/R347 protein level in retinal membrane protein extract | p value (wt/R347 protein level in whole retina protein extract) | p value (wt/R347 protein level in retinal membrane protein extract) |
| --- | --- | --- | --- | --- | --- |
| Actb | 9.0 |  |  | 0.84 | 0.152 |
| Calr | 7.6 | 1.74 | 0.43 | 0.019 | 0.087 |
| Canx | 7.4 | 1.11 | 1 | 0.464 | 0.984 |
| Cav1 | 5.5 |  | 0.5 |  | 0.001 |
| Cltc | 6.8 | 0.93 | 0.72 | 0.227 | 0.008 |
| Cntnap1 | 4.1 |  | 1.16 |  | 0.465 |
| Eef1g | 7.8 | 0.82 |  | 0.181 |  |
| Gnai2 | 7.0 | 2.26 | 0.64 | 0.678 | 0.000 |
| Gnai3 | 4.4 |  | 1.19 |  | 0.379 |
| Gnb1 | 11.0 | 2.42 | 2.64 | 0.000 | 0.000 |
| Hnrnpa0 | 6.0 | 0.81 |  | 0.096 |  |
| Hspa5 | 7.1 | 0.75 | 1.22 | 0.058 | 0.363 |
| Iqgap2 | 1.6 | 1.22 |  | 0.220 |  |
| Ktn1 | 5.7 |  | 1.29 |  | 0.248 |
| Lrrc59 | 5.2 | 0.83 | 0.73 | 0.572 | 0.011 |
| Nckap1 | 5.6 | 1.95 |  | 0.007 |  |
| Nme1 | 7.1 | 0.49 | 4.02 | 0.300 | 0.002 |
| Nod2 | 0.0 |  | 1.13 |  | 0.518 |
| Pdia6 | 5.6 | 0.77 | 0.83 | 0.023 | 0.400 |
| Pgrmc1 | 7.4 | 0.73 | 0.46 | 0.074 | 0.003 |
| Ppp2r4 | 5.9 | 0.7 |  | 0.003 |  |
| Rab10 | 5.2 | 0.42 | 0.89 | 0.036 | 0.383 |
| Rac1 | 6.2 | 0.29 | 1.66 | 0.000 | 0.016 |
| Rap1a | 5.0 | 0.92 | 0.82 | 0.400 | 0.432 |
| Rap1gds1 | 5.8 | 0.49 |  | 0.000 |  |
| Sfpq | 5.7 | 1.2 |  | 0.076 |  |
| Sod1 | 7.5 | 0.86 |  | 0.652 |  |
| Tmem33 | 4.6 |  | 1.57 |  | 0.177 |
| Vapb | 4.4 |  | 0.99 |  | 0.861 |

| **b** |  |  |  |  |  |
| --- | --- | --- | --- | --- | --- |
| Gene | Expression (log2 of  FPKM/RPKM) | wt/R347 protein level in whole retina protein extract | wt/R347 protein level in retinal membrane protein extract | p value (wt/R347 protein level in whole retinal protein extract) | p value (wt/R347 protein level in retinal membrane protein extract) |
| Actb | 9.0 |  |  | 0.84 | 0.152 |
| Actg1 | 8.4 | 1 |  | 0.835 |  |
| Eno1 | 9.5 | 1.54 | 1.79 | 0.032 | 0.003 |
| Gnb5 | 8.9 | 1.51 |  | 0.500 |  |
| Pde6c | 4.7 |  | 0.79 |  | 0.235 |
| Prdx2 | 8.2 | 0.92 |  | 0.679 |  |
| Rho | 13.5 | 6.14 | 44.98 | 0.001 | 0.000 |
| Rhoa | 6.0 | 0.79 | 1.23 | 0.095 | 0.247 |
| Sag | 12.2 | 2.11 | 1.48 | 0.000 | 0.099 |

**Supplementary Table S5** Alterations in protein levels between R347 and wt retinas in the subset of Rac1 interactome detected in this study. The mouse Rac1 interactome was generated in InnateDB [31]. Part **a** Wt versus R347 retinal protein levels were determined for 29 proteins of the interactome using LC-MS/MS (n=4) and p values calculated (ANOVA). Part **b** Wt versus R347 retinal protein levels for previously identified photoreceptor outer segment Rac1 interactors [33] detected in our study using LC-MS/MS (n=4) were determined and p values calculated (ANOVA). Relative expression levels (log2 scale of FPKM/RPKM) were added from the mouse retinal transcriptome library (Supplementary Table S1) [11, 23].

**Supplementary Table S6**

| Folr1  (XM_006507360.1) | Folr1F CGGCTGTCTCCTGGAATGAA  Folr1R AGCACAGGAAAAAGCCCAGA |
| --- | --- |
| Plxna4  (NM_175750.3) | Plxna4F ACTGCCCGGCTAGTCCC  Plxna4R CGGCGAGTACGTTGGGG |
| Tmed5  (XM_006535240.1) | Tmed5F AGGAAGGATGCGAAACCCAG  Tmed5R CTGTCGATCTCTGCCTGGAC |
| Ttc21b  (NM_001047604.1) | Ttc21bF ACACCTTCACCGAAATCGCA  Ttc21R TCTTCTCCAGATCCTCGGCT |
| Rac1  (NM_009007.2) | Rac1F CCGCAGACAGACGTGTTCTTA  Rac1R GGTGATGGGAGTCAGCTTCT |
| Actb  (NM_007393.3) | ActbF AGAGCAAGAGAGGCATCC  ActbR TCATTGTAGAAGGTGTGGTGC |

**Supplementary Table S6** Sequence of PCR primers used in this study.
